# An Immunomagnetic Bead-Based Sandwich ELISA for the Detection of *Brucella* in Milk

**DOI:** 10.3390/ani16091330

**Published:** 2026-04-27

**Authors:** Gaowa Wudong, Qing Lu, Danyu Zhao, Yong Shi, Yimeng Cui, Yaping Jin, Dong Zhou, Aihua Wang

**Affiliations:** 1 College of Veterinary Medicine, Northwest A&F University, Xianyang 712100, China; wudonggaowaa@163.com (G.W.);; 2 Key Laboratory of Animal Biotechnology, Ministry of Agriculture, Northwest A&F University, Xianyang 712100, China

**Keywords:** *Brucella*, immunomagnetic beads, sandwich ELISA

## Abstract

Brucellosis is an important zoonotic disease caused by *Brucella* spp., posing significant impacts on animal health, livestock production, and public health. Infected dairy animals can shed the pathogen in milk, making milk a noninvasive and readily accessible sample for disease surveillance. Therefore, detection of *Brucella* in milk can be used for monitoring brucellosis in dairy herds. In this study, polyclonal antibodies were produced by immunizing camels with lipopolysaccharide (LPS) derived from *Brucella abortus* vaccine strain 19 (A19). These antibodies were subsequently conjugated to magnetic beads to develop immunomagnetic beads (IMBs). The IMBs were combined with a sandwich ELISA for the enrichment and detection of *Brucella* in milk samples. The IMB-sELISA showed a limit of detection (LOD) of 1 × 10^4^ CFU/mL. The method reduced matrix interference and showed satisfactory analytical performance. This approach can be applied for on-farm sample screening and herd-level surveillance to support the diagnosis and control of brucellosis in dairy animals.

## Simple Summary

Brucellosis is a disease that can spread from animals to humans and causes major losses in dairy farming. Infected animals may release the bacteria into milk, making milk an easy sample for testing without harming the animals. This study aimed to develop a simple and effective way to detect these bacteria in milk. Researchers produced specific substances in camels that can recognize the bacteria and attached them to tiny magnetic particles to help collect the bacteria from milk. A follow-up test was then used to confirm their presence. The method was able to detect low levels of bacteria and worked well in commercial milk samples. This approach provides a practical tool for rapid screening on farms, helping farmers and veterinarians identify infection early, control disease spread, and protect both animal productivity and public health.

## 1. Introduction

Brucellosis is an important zoonotic disease caused by *Brucella* spp. [1] and has significant impacts on animal health, livestock production, and public health. In dairy animals, infection with *Brucella* is commonly associated with abortion, infertility, and reduced productivity, resulting in substantial and persistent economic losses at the herd level. Therefore, accurate and reliable diagnostic methods are essential for the effective surveillance and control of brucellosis. Serological assays, such as the Rose Bengal Test (RBT), complement fixation test (CFT), and enzyme-linked immunosorbent assay (ELISA), are widely used for diagnosis and surveillance and are recommended by the World Organisation for Animal Health (WOAH).

However, in regions where vaccination programs are implemented, some animals may develop persistent antibodies following immunization [2,3,4], which complicates the differentiation between natural infection and vaccine-induced immune responses [5]. Therefore, reliance on serological assays alone is insufficient for accurate diagnosis, and diagnostic approaches incorporating antigen detection are needed to improve detection accuracy in dairy animals.

In dairy herd-level brucellosis surveillance, the effectiveness of diagnostic strategies is closely related to the choice of sampling method. Compared with invasive blood sampling, milk sampling is more convenient, readily accessible, and suitable for repeated collection, making it well suited for monitoring *Brucella* infection in dairy cattle populations [6]. In addition, milk-based testing can be performed using bulk milk samples, which are appropriate for routine herd-level screening, simplifying analytical procedures. The Milk Ring Test (MRT), recommended by the WOAH, is a commonly used screening method for milk samples. However, this assay relies on subjective visual interpretation and has limited diagnostic sensitivity [7]. These limitations highlight the need for rapid, sensitive, and reliable detection methods specifically designed for milk samples to support herd-level surveillance and control of brucellosis.

Currently, direct detection of *Brucella* primarily relies on bacterial isolation and culture, as well as molecular methods such as PCR [8]. Bacterial isolation and culture are considered the gold standard for the diagnosis of brucellosis [9]. However, these methods require high-level biosafety facilities, involve prolonged incubation periods, and demand strict operational procedures, which limit their applicability in routine testing [9]. Although PCR-based methods offer high sensitivity and specificity, they typically depend on specialized instrumentation and complex sample preparation, making them less suitable for field application and large-scale surveillance. In addition, the low abundance of target bacteria and the complex composition of milk samples may interfere with detection performance. Therefore, effective sample pretreatment is essential for improving the accuracy of microbial detection in milk.

Immunomagnetic separation (IMS) is an efficient sample pretreatment technique with high specificity and separation efficiency, enabling rapid enrichment of target bacteria while reducing interference from non-target microorganisms and sample matrices [10,11]. IMS has been widely integrated with various detection methods, including ELISA [12,13], PCR [14,15,16], fluorescence lateral flow immunoassay [17], and flow cytometry [18,19], thereby improving detection sensitivity and reliability. IMS performance largely depends on the properties of the antibodies used for target recognition. Antibodies derived from camels exhibit good stability, strong antigen-binding capacity, and high tolerance to complex environments, making them advantageous for diagnostic applications [20,21]. These characteristics also support their effective use in detecting targets in complex sample matrices such as milk.

In this study, we aimed to develop a method for detecting *Brucella* in milk by combining immunomagnetic separation with immunoassay. This approach is expected to enhance detection efficiency and accuracy and has potential applications in herd-level surveillance and control of brucellosis in dairy animals.

## 2. Materials and Methods

### 2.1. Materials

Mouse polyclonal antibodies against *Brucella abortus* vaccine strain 19 (A19) were maintained in our laboratory, along with *Salmonella*, *Staphylococcus aureus*, *Escherichia coli* F17, and *Streptococcus agalactiae* strains. rProtein A/G Beads 4FF were purchased from Smart Lifesciences (Changzhou, China). Magnetic separation racks and carboxyl functionalized magnetic beads were obtained from Beyotime Biotechnology (Shanghai, China). Commercial milk samples (pasteurized) were purchased from a local supermarket. Raw milk samples were collected from a dairy farm in Ningxia, China, where an increased incidence of abortions was observed during the sampling period, and brucellosis had been confirmed. All experiments involving the A19 strain were conducted in a biosafety level 2 laboratory. All procedures were performed in a certified Class II biological safety cabinet in accordance with institutional biosafety regulations.

### 2.2. Preparation of Polyclonal Antibodies Against LPS

The A19 strain was inoculated into tryptic soy broth (TSB) and incubated at 37 °C with agitation for 72 h. Following heat inactivation, the bacterial biomass was harvested via centrifugation at 12,000× *g*. LPS was extracted using a commercial LPS extraction kit (IntronBio, Seongnam-si, Republic of Korea), and its concentration was determined via the phenol–sulfuric acid method. For immunization, 2 mL of LPS at a concentration of 1.5 mg/mL was emulsified with an equal volume of Freund’s complete adjuvant and administered to camels. Subsequent booster immunizations were performed at two-week intervals using LPS at the same concentration emulsified with Freund’s incomplete adjuvant. Six days after the third immunization, approximately 200 mL of blood was collected, and serum was obtained via centrifugation for antibody titer evaluation. Serum collected prior to immunization served as a negative control. All serum samples were filtered through a 0.45-µm membrane and incubated with Protein A/G Beads 4FF overnight at 4 °C, and then purified. Samples from each purification step were analyzed by means of SDS-PAGE.

### 2.3. Preparation of Immunomagnetic Beads (IMBs)

A 100 μL suspension of magnetic beads was transferred into a centrifuge tube and subjected to magnetic separation for 10 s to remove the supernatant, followed by three washes with 500 μL of activation buffer. Subsequently, 100 μL of freshly prepared EDC solution and 100 μL of NHS solution were added, and the mixture was incubated under gentle rotation at 25 °C for 60 min to activate the bead surface. After magnetic separation for 10 s to remove the supernatant, polyclonal antibodies (25, 50, 100, 150, and 200 μg) were added, followed by incubation under gentle rotation at 25 °C for 2 h to allow conjugation. The coupling efficiency was calculated using the following equation: Coupling rate (%) = (1 − C_2_/C_1_) × 100, where C_1_ and C_2_ represent the concentrations of the polyclonal antibody before and after conjugation, respectively. All experiments were performed in triplicate, and the results are presented as mean ± SD.

Upon completion of the reaction, the beads were magnetically separated for 10 s to remove the supernatant and washed three times with 500 μL of reaction buffer, yielding IMBs. To block unreacted sites, 500 μL of blocking solution was added, followed by incubation at 25 °C for 2 h. After magnetic separation for 10 s and three washes with 500 μL of reaction buffer, the beads were resuspended in 100 μL of protective solution and stored at 4 °C.

### 2.4. Optimization of the IMS Procedure

To evaluate the performance of the IMBs, 100 μL of IMBs were added to a *Brucella* suspension (1 × 10^5^ CFU/mL) and incubated under gentle rotation at 37 °C for 1 h. Following incubation, the IMBs were collected using a magnetic separator. Both the supernatant and the IMB–bacteria complexes were subjected to tenfold serial dilutions and subsequently plated onto Tryptic Soy Agar (TSA) plates. After incubation for 72 h, colonies were counted. The capture efficiency (CE) of the IMBs was calculated using the following equation: CE (%) = (1 − N_2_/N_1_) × 100, where N_1_ represents the total CFU of *Brucella* in the sample and N_2_ represents the CFU detected in the supernatant.

To enhance the separation performance of the IMBs, three key parameters—IMB dose, incubation time, and magnetic separation time—were systematically optimized. The optimal conditions were determined based on the highest CE.

### 2.5. Sample Analysis of IMBs

To evaluate the performance of the IMBs in different sample matrices, commercial milk and raw milk samples were spiked with *Brucella* to a final concentration of 1 × 10^5^ CFU/mL, with PBS serving as the control. After the addition of IMBs, the samples were incubated under gentle rotation at 37 °C for 1 h, followed by magnetic separation to collect the bead–bacteria complexes. The complexes were then washed three times with PBS, serially diluted and plated onto TSA plates. After incubation for 72 h, colony counts were recorded, and the capture efficiency of the IMBs was calculated for each sample type.

### 2.6. Establishment of the Sandwich ELISA

A sandwich ELISA was established using camel polyclonal antibodies as the capture antibody and mouse polyclonal antibodies as the detection antibody. The optimal working concentrations of both antibodies were initially determined by means of checkerboard titration. Camel polyclonal antibodies at concentrations of 2.5, 5, 10, and 20 μg/mL were coated onto ELISA plates, followed by blocking with 3% skim milk for 1 h and washing three times with PBST. Heat-inactivated *Brucella* (1 × 10^7^ CFU/mL) and *Escherichia coli* (1 × 10^7^ CFU/mL) were added as the positive and negative samples, respectively, and incubated at 37 °C for 1 h. After washing, mouse polyclonal antibodies diluted at 1:50, 1:100, 1:200, or 1:300 were added and incubated at 37 °C for 1 h. After washing with PBST, goat anti-mouse IgG-HRP was added and incubated at 37 °C for 1 h. Subsequently, TMB substrate was added and incubated for 15 min in the dark. The reaction was stopped with 2 M H_2_SO_4_, and the absorbance was measured at 450 nm. The optimal coating concentration of camel polyclonal antibodies and the optimal dilution of mouse polyclonal antibodies were determined based on the highest positive-to-negative (P/N) ratio.

Based on the above optimized antibody conditions, the dilution of goat anti-mouse IgG-HRP and the incubation time of bacteria with the coating antibody were further optimized. Goat anti-mouse IgG-HRP was tested at dilutions of 1:5000, 1:8000, 1:10,000, and 1:15,000, and the incubation time of bacteria with the coating antibody was evaluated at 30, 45, 60, and 90 min. The optimal parameters were selected based on the highest P/N ratio.

To determine the cut-off value of the sandwich ELISA, thirty milk samples confirmed to be negative by PCR were analyzed. The cut-off value was calculated as the mean OD_450_ of the negative samples plus three standard deviations (mean OD_450_ + 3 × SD). This cut-off was subsequently applied to classify samples, with OD_450_ values greater than the cut-off considered positive and those equal to or lower than the cut-off considered negative.

### 2.7. Specificity and Repeatability Evaluation of the IMB-sELISA

To assess the analytical specificity of the IMB-sELISA, suspensions of *Brucella*, *Salmonella*, *Staphylococcus aureus*, *E. coli* F17, and *Streptococcus agalactiae* at a concentration of 1 × 10^6^ CFU/mL were prepared and analyzed. Each bacterial strain was enriched using IMBs, eluted, and subsequently subjected to the sandwich ELISA. The specificity of the IMB-sELISA was assessed by comparing the OD_450_ signal intensities obtained for the different bacterial species.

To evaluate the repeatability of the IMB-sELISA, both intra- and inter-assay variability were assessed. Intra-assay variability was determined by analyzing three replicates of *Brucella* samples within a single assay run, whereas inter-assay variability was evaluated by testing the samples with reagents from three independent batches.

### 2.8. Preliminary Evaluation of the Detection Range of IMB-sELISA

The IMB-sELISA was evaluated using serial concentrations of *Brucella* ranging from 1 × 10^1^ to 1 × 10^7^ CFU/mL to preliminarily determine its detectable range. The LOD of the IMB-sELISA was ultimately established by comparing the measured OD_450_ values with the predetermined cut-off value.

### 2.9. Application of the IMB-sELISA in Milk Samples

To evaluate the applicability of the IMB-sELISA for milk sample analysis, a total of 80 raw milk samples were collected from dairy cows and subjected to testing. The samples were initially heat-treated and subsequently centrifuged at 5000 × *g* for 10 min to remove the upper cream layer. The processed samples were then analyzed using the IMB-sELISA, and all results were further validated by means of PCR. The PCR assay employed primers targeting the *Brucella* bcsp31 gene [22].

## 3. Results

### 3.1. Preparation and Purification of Camel Polyclonal Anti-LPS Antibodies

**Figure 1:**
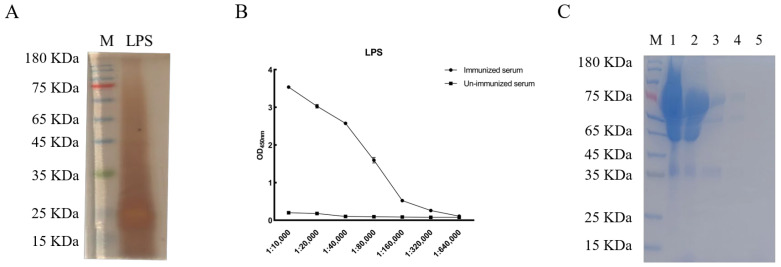
Preparation of LPS Polyclonal Antibodies. (**A**) Silver staining analysis of purified LPS. (**B**) Antibody titer against LPS in camel serum. (**C**) Purification of camel polyclonal antibodies against LPS. Lane M: protein molecular weight marker; lanes 1–2: flow-through fractions collected during purification; lanes 3–5: elution fractions containing purified antibodies.

LPS derived from A19 was successfully extracted and verified via silver staining (Figure 1A). The purified LPS was used to immunize a male Bactrian camel, and three immunizations were administered in total. Serum samples collected on day 6 after the third immunization showed that the antibody titer reached 1:160,000 (Figure 1B), indicating a robust humoral immune response. Polyclonal antibodies were subsequently purified using rProtein A/G Beads 4FF. SDS-PAGE analysis of the eluted fractions showed a prominent antibody band with minimal nonspecific proteins, indicating successful purification of camel anti-LPS polyclonal antibodies (Figure 1C).

### 3.2. Optimization and Performance of the IMS

**Figure 2:**
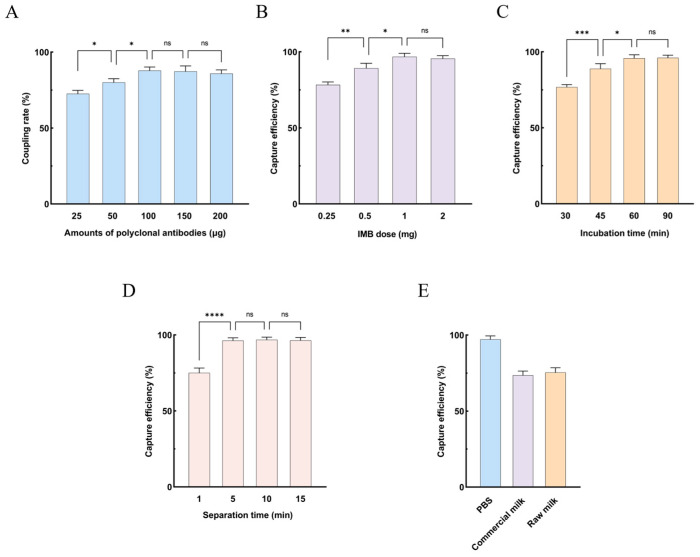
Optimization of IMS and milk sample analysis. (**A**) Optimal antibody coupling amount. (**B**) Optimal IMB dose. (**C**) Optimal incubation time. (**D**) Optimal magnetic separation time. (**E**) Recovery rate of IMBs in PBS and milk samples. Data are presented as mean ± SD (n = 3). * *p* < 0.05; ** *p* < 0.01; *** *p* < 0.001; **** *p* < 0.0001; ns, not significant.

IMBs were prepared using increasing amounts of camel polyclonal antibodies to optimize the coupling efficiency between antibodies and magnetic beads. The coupling efficiency increased markedly with increasing antibody amount and reached a maximum at 100 μg, after which it plateaued (Figure 2A). Accordingly, 100 μg of camel polyclonal antibodies was selected as the optimal amount for IMB preparation.

To further enhance the capture performance of the IMBs, the amount of magnetic beads was optimized. The capture efficiency increased as the bead amount increased and reached the highest value at 1 mg (Figure 2B). In addition, both the incubation time and magnetic separation time were evaluated. The capture efficiency reached a maximum at 1 h of incubation (Figure 2C), and a magnetic separation time of 5 min was optimal (Figure 2D).

### 3.3. Performance of IMBs in Different Sample Matrices

The performance of IMBs was evaluated in different sample matrices (PBS, commercial milk, and raw milk) at a *Brucella* concentration of 1 × 10^5^ CFU/mL. The recovery rates ranged from 94.5% to 98.8% in PBS, 70.2% to 75.4% in commercial milk, and 72.5% to 78.6% in raw milk (Figure 2E).

### 3.4. Establishment of the IMB-sELISA

**Figure 3:**
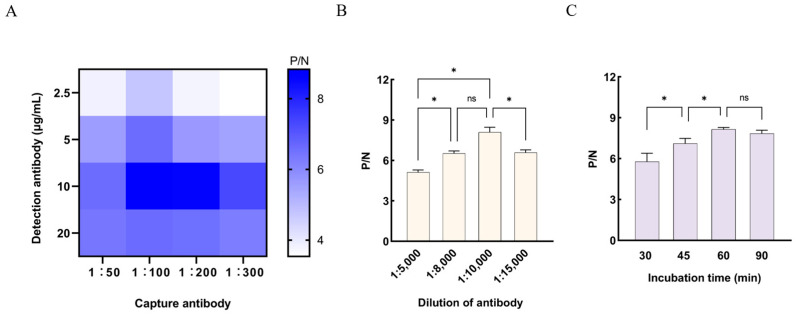
Establishment and Optimization of the IMB-sELISA. (**A**) Determination of the optimal capture antibody concentration and optimal detection antibody dilution using the checkerboard method. (**B**) Optimal dilution of the secondary antibody. (**C**) Optimal incubation time. Data are presented as mean ± SD (n = 3). * *p* < 0.05; ns, not significant.

The coating concentration of the capture antibody and the dilution of the detection antibody were optimized using a checkerboard titration assay. As shown in Figure 3A, the highest P/N ratio was obtained when the capture antibody concentration was 10 μg/mL and the detection antibody dilution was 1:100. This combination was therefore used in subsequent experiments. The dilution of goat anti-mouse IgG-HRP and the bacterial incubation time were then further optimized. As illustrated in Figure 3B, a dilution of 1:10,000 produced the highest P/N ratio and was therefore identified as the optimal working dilution for goat anti-mouse IgG-HRP. In addition, an incubation time of 60 min resulted in the highest P/N ratio (Figure 3C) and was selected as the optimal incubation time.

To establish the cut-off value for the IMB-based sandwich ELISA, thirty negative milk samples were analyzed. The mean OD_450_ value of these samples was 0.3142, with a standard deviation of 0.022. Following the commonly applied criterion of “mean + 3SD,” the cut-off value was calculated to be 0.3802. Samples with OD_450_ values greater than 0.3802 were classified as positive, whereas those with OD_450_ values ≤ 0.3802 were classified as negative.

### 3.5. Specificity and Repeatability of the IMB-sELISA

**Figure 4:**
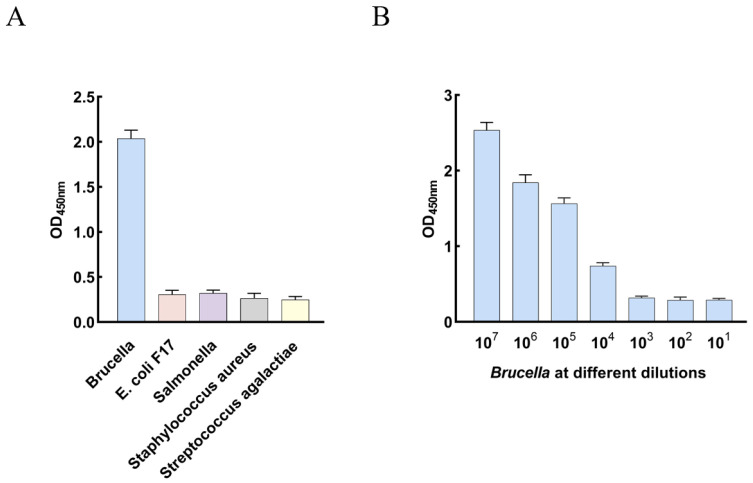
Specificity and limit of detection of the IMB-sELISA. (**A**) Specificity testing of IMB-sELISA. (**B**) Limit of detection for *Brucella* using IMB-sELISA.

To assess the specificity of the IMB-sELISA, *Brucella*, *Salmonella*, *Staphylococcus aureus*, *E. coli* F17, and *Streptococcus agalactiae* were tested. As shown in Figure 4A, only *Brucella* generated a positive signal, while no cross-reactivity was observed with the other bacterial species, indicating high specificity of the method.

To further evaluate the repeatability of the IMB-sELISA, both intra-assay and inter-assay variations were analyzed. As presented in Table 1, the intra-assay coefficients of variation (CVs) ranged from 5.3% to 8.4% (median 6.8%), while the inter-assay CVs ranged from 6.5% to 10.2% (median 9.1%). All CVs were within acceptable limits, demonstrating that the assay exhibits good repeatability.

### 3.6. Limit of Detection of the IMB-sELISA

To determine the LOD of the IMB-sELISA, inactivated *Brucella* suspensions from 1 × 10^1^ to 1 × 10^7^ CFU/mL were analyzed. The results demonstrated that the assay could detect *Brucella* at a concentration of 1 × 10^4^ CFU/mL (Figure 4B).

### 3.7. Application of the IMB-sELISA in Raw Milk Samples

The IMB-sELISA was applied to analyze 80 raw milk samples collected from dairy cows on a farm with an increased abortion rate. Among these samples, two tested positive, while the remaining 78 were negative. All samples were subsequently re-examined using PCR, and the results were consistent with those obtained using the IMB-sELISA (Table 2).

## 4. Discussion

Brucellosis is one of the most widespread zoonotic bacterial diseases worldwide [23]. In dairy animals, infected individuals shed *Brucella* through aborted materials, vaginal secretions [24], and milk [25], thereby facilitating disease transmission. Among these, milk is not only a major route of bacterial shedding but also a convenient, noninvasive sample that can be collected repeatedly, making it particularly suitable for herd-level surveillance [26]. Such surveillance is critical for effective control of brucellosis, and routine self-monitoring at the farm level is therefore warranted. Against this background, the development of sensitive and reliable milk-based detection methods is essential for surveillance and early disease control.

The results of this study suggest that the IMB-sELISA system enables effective enrichment and detection of *Brucella* in milk samples and may provide a useful approach for herd-level monitoring in dairy animals. Compared with serological assays that are susceptible to interference from vaccine-induced antibodies [27,28], this method targets bacterial cells directly and may help to reduce some limitations associated with conventional antibody-based diagnostics in vaccinated populations.

The IMB-sELISA system offers several technical advantages. First, immunomagnetic beads enable efficient *Brucella* enrichment from milk, reducing interference from milk components and non-target microorganisms, thus improving detection reliability. Previous studies have shown that immunomagnetic bead-based methods enhance *Brucella* detection performance, particularly in complex matrices, improving sensitivity [29,30]. Second, polyclonal antibodies raised against LPS from the A19 strain were used as recognition elements. LPS, as a major surface antigen, is highly exposed [31], facilitating the detection of intact bacterial cells. Additionally, the O-polysaccharide structure of smooth-type *Brucella* (including *B. abortus*, *B. melitensis*, and *B. suis*) is highly conserved [28], enabling potential cross-reactivity across multiple smooth-type strains. Overall, compared with conventional culture methods and PCR, IMB-sELISA strikes a balance between sensitivity, specificity, and operational simplicity. The method requires minimal instrumentation and shows promise for milk sample screening.

In practical applications, this method can be integrated into routine farm monitoring programs, with screening carried out by trained farm personnel, veterinarians, or diagnostic laboratories. For positive samples, PCR confirmation should be followed by immediate isolation of the infected animals, prompt reporting to relevant authorities, and the initiation of control measures to minimize the risk of disease transmission.

Beyond veterinary applications, this method may also have potential value for diagnosing bacteremic brucellosis in humans. In regions where “Brucellosis-Free” status has not yet been achieved and human morbidity remains high, reliable detection of bacteremia is important for clinical management. The ability of this method to reduce matrix interference and directly detect bacterial cells may help identify *Brucella* in complex clinical samples, although further validation in human specimens is still required.

Despite these advantages, several limitations must be considered. The detection limit of 1 × 10^4^ CFU/mL may restrict detection in samples with low bacterial loads, particularly during early infection. Therefore, this method is more suitable as a screening tool than a confirmatory diagnostic approach. Negative results in suspected cases should be interpreted with caution and verified using more sensitive methods like PCR. In addition, as the antibodies were raised against smooth-type LPS, whose antigenicity is primarily determined by the O-polysaccharide, the method is mainly applicable to smooth-type *Brucella*, whereas rough-type strains (e.g., *B. canis*), lacking the O-polysaccharide, are unlikely to be effectively recognized. Batch-to-batch variability in polyclonal antibodies [32] may also affect reproducibility and scalability. To ensure long-term consistency, standardized immunization and purification procedures, together with key quality indicators, should be used to define and evaluate acceptance criteria for each antibody batch. Moreover, the relatively small sample size and the limited number of positive samples, coupled with the absence of comparison with culture-based reference methods, may limit the generalizability of the findings.

To address these limitations, future studies should expand the sample size and include a wider range of *Brucella* strains, encompassing both additional smooth-type species and rough-type strains, along with comparisons to culture-based reference methods. In terms of optimization, recombinant antigens could be explored as alternative immunogens to replace LPS, while monoclonal antibodies or camelid-derived VHH antibodies may serve as recognition elements to improve assay consistency and batch-to-batch reproducibility. Additionally, signal amplification strategies, such as biotin–streptavidin systems, may enhance sensitivity [33,34]. Finally, the antibody-based recognition strategy and immunomagnetic enrichment approach described here may provide a basis for developing portable formats, such as lateral flow assays, for rapid field detection.

## 5. Conclusions

In conclusion, this study developed an immunomagnetic bead-based sandwich ELISA method for the enrichment and detection of *Brucella* in milk samples. The method demonstrated acceptable performance in terms of sensitivity, reproducibility, and agreement with PCR results under the experimental conditions. However, further optimization and validation with a larger number of field samples are required. Overall, IMB-sELISA shows potential as a practical approach for milk-based screening and may contribute to herd-level surveillance of brucellosis in dairy animals.

## Figures and Tables

**Table 1:** Assessment of Repeatability for IMB-sELISA.

Items	CV Range (%)	Median CV (%)
Intra-assay variation	5.3–8.4	6.8
Inter-assay variation	6.5–10.2	9.1

**Table 2:** Comparison of IMB-sELISA and PCR for *Brucella* Detection in Raw Milk Samples.

Method	Positive Samples	Negative Samples	Positive Rate	Agreement Rate
IMB-sELISA	2	78	2.5%	100% *
PCR	2	78	2.5%	

* The 100% agreement rate is based on 80 samples and should be further validated with a larger sample size.

## Data Availability

All data generated or analyzed during this study are available from the corresponding author upon reasonable request.

## Abbreviations

IMBs	Immunomagnetic beads
IMS	Immunomagnetic separation
LPS	Lipopolysaccharide
LOD	Limit of detection
MRT	Milk Ring Test
TSA	Tryptic Soy Agar
TSB	Tryptic soy broth
WOAH	World Organisation for Animal Health
